# IDENTIFYING GENDER-BASED DIFFERENCES IN CHARACTERISTICS OF OLDER ADULT CANNABIS USERS

**DOI:** 10.1093/geroni/igae098.3151

**Published:** 2024-12-31

**Authors:** Juliamaria Coromac-Medrano, Elizabeth Anquillare, Adrianna Neiderman, Rachel Thayer

**Affiliations:** University of Colorado Colorado Springs, Colorado Springs, Colorado, United States; University of Colorado Colorado Springs, Colorado Springs, Colorado, United States; University of Colorado Colorado Springs, Colorado Springs, Colorado, United States; University of Colorado Colorado Springs, Colorado Springs, Colorado, United States

## Abstract

As cannabis use continues to rise among older adults, understanding different patterns of cannabis use is essential for interpreting health outcomes and treatment expectations for this population. This study explored gender differences in age of onset, frequency of use, primary methods of cannabis ingestion, and scores on substance use disorder and cognitive questionnaires between male and female older adult cannabis users (OACU). This sample included 52 OACU ages 60+ (Mage=67.98, SD=5.77; 50%=female; 90%=White). Chi-square analyses and independent samples t-tests were performed to identify gender differences in patterns of cannabis use. The percentage of men who endorsed primarily inhalation methods (76%) was significantly greater than women (46%), and the percentage of women who endorsed primarily consuming edibles (54%) was significantly greater than men (24%), χ2(1, N=49)=4.69, p<.05. Men were more likely to use cannabis on more days of the previous week (M=5.42, SD=2.42) compared to women (M=3.88, SD=2.83), t(50)=2.11, p<.05. Men also generated significantly greater scores on the Cannabis Use Disorder Identification Test-Revised (CUDIT-R) compared to women (Mscore=12.85, SD=6.75 and Mscore=8.96, SD=5.85, respectively), t(50)=2.22, p<.05. Women (Mscore=195.48, SD=46.88) reported significantly greater cognitive self-efficacy compared to men (Mscore=168.35, SD=44.54), t(49)=-2.18, p<.05. There was no difference regarding the age of first ever cannabis use between men (Mage=22) and women (Mage=26). These findings elucidate several differences in use patterns between male and female OACU. Future research can explore additional gender differences to better inform clinical teams about the individual characteristics of an older adult cannabis user.

